# IUC24428-85 Real-world effectiveness of ipilimumab plus nivolumab in second-line in patients with metastatic clear cell renal cell carcinoma (mccRCC) based on prior receipt of PD-1/L1 inhibitors

**DOI:** 10.1093/oncolo/oyaf248.046

**Published:** 2025-08-29

**Authors:** Z I Ozay, Y Jo, G Gebrael, M Ostrowski, E Murdock, S Jamie-Casas, M Zugman, N Agarwal, S Pal, U Swami

**Affiliations:** Huntsman Cancer Institute, United States; Huntsman Cancer Institute, United States; Huntsman Cancer Institute, United States; Huntsman Cancer Institute, United States; Huntsman Cancer Institute, United States; City of Hope, United States; City of Hope, United States; Huntsman Cancer Institute, United States; City of Hope, United States; Huntsman Cancer Institute, United States

## Abstract

**Background:**

Previous phase 3 studies have established that the addition of PD-1/L1 inhibitors (PD-1/L1i) to a tyrosine kinase inhibitor (TKI) after progression on prior anti-PD-1/L1 therapy does not yield additional benefit over single-agent TKI [PMID: 37290461, 39284329]. However, limited data are available from 2 phase 2 trials regarding the use of ipilimumab plus nivolumab (ipi-nivo) after prior PD-1/L1 therapies [PMID: 36328377, 37844597]. Herein, we aimed to assess the real-world effectiveness of ipi-nivo in second-line (2 L) in patients with mccRCC after prior treatment with PD-1/L1i.

**Methods:**

This study used the nationwide Flatiron Health electronic health record (EHR)-derived de-identified database to extract patient-level data. Eligibility criteria: diagnosis of mccRCC and receipt of ipi-nivo in 2 L. Patients treated with an ipilimumab-containing regimen in the 1 L or those involved in clinical trials were excluded. The primary endpoints were real-world time to next therapy (rwTTNT) and overall survival (rwOS) from the start of 2 L treatment and were summarized using median survival and 95% confidence interval (CI) via the Kaplan–Meier method.

**Results:**

Of 12,285 patients with mccRCC, 196 met the eligibility criteria, including 63 who received PD-1/L1i as 1 L therapy. The median age of the cohort was 65 years, with 143 (73.0%) male and 133 (68.9%) White non-Hispanic patients. The median rwTTNT with 2 L ipi-nivo was 8.60 months (95% CI, 3.73-30.67) for patients without prior PD-1/L1i therapy and 6.67 months (95% CI, 3.10-24.83) for those with prior PD-1/L1i therapy. Median rwOS was 28.93 months (95% CI, 12.43-42.43) for patients without prior PD-1/L1i therapy and 24.83 months (95% CI, 9.10-47.27) for those with prior PD-1/L1i therapy. Results stratified per IMDC category will be presented in the meeting.

**Conclusions:**

In this real-world patient population, ipi-nivo as 2 L Rx in mccRCC retained effectiveness (compared to historically reported data with other agents in salvage therapy settings), regardless of prior PD-1/L1i exposure. However, given modest rwTTNT, novel therapies or combination regimens are needed. Limitations to this study include selection bias, lack of toxicity data as the cause of drug discontinuation, and randomized controls.

